# Chronic inflammation of middle ear cholesteatoma promotes its recurrence via a paracrine mechanism

**DOI:** 10.1186/s12964-020-00690-y

**Published:** 2021-02-24

**Authors:** Matthias Schürmann, Felix Oppel, Senyao Shao, Verena Volland-Thurn, Christian Kaltschmidt, Barbara Kaltschmidt, Lars-Uwe Scholtz, Holger Sudhoff

**Affiliations:** 1 Department of Otolaryngology, Head and Neck Surgery, Medical School OWL Campus Klinikum Bielefeld, Bielefeld University, Teutoburger Str. 50, 33604 Bielefeld, Germany; 2 Department of Cell Biology, Bielefeld University , 33619 Bielefeld, Germany

**Keywords:** Cholesteatoma, Inflammation, TLR4, Stem cells, Cholesteatoma recurrence

## Abstract

**Background:**

Cholesteatoma disease is an expanding lesion in the middle ear. Hearing loss and facial paralysis alongside with other intracranial complications are found. No pharmaceutical treatment is available today and recurrence after surgical extraction occurs. We investigated possible TLR4-based mechanisms promoting recurrence and explore possible treatments strategies.

**Methods:**

We isolated fibroblasts and epidermal stem cells from cholesteatoma tissue and healthy auditory canal skin. Subsequently, their expression under standard culture conditions and after stimulation with LPS was investigated by RT-qPCR. Cell metabolism and proliferation were analysed upon LPS treatment, with and without TLR4 antagonist. An indirect co-culture of fibroblasts and epidermal stem cells isolated from cholesteatoma tissue was utilized to monitor epidermal differentiation upon LPS treatment by RT-qPCR and immunocytochemistry.

**Results:**

Under standard culture conditions, we detected a tissue-independent higher expression of IL-1β and IL-8 in stem cells, an upregulation of KGF and IGF-2 in both cell types derived from cholesteatoma and higher expression of TLR4 in stem cells derived from cholesteatoma tissue. Upon LPS challenge, we could detect a significantly higher expression of IL-1α, IL-1β, IL-6 and IL-8 in stem cells and of TNF-a, GM-CSF and CXCL-5 in stem cells and fibroblasts derived from cholesteatoma. The expression of the growth factors KGF, EGF, EREG, IGF-2 and HGF was significantly higher in fibroblasts, particularly when derived from cholesteatoma. Upon treatment with LPS the metabolism was elevated in stem cells and fibroblasts, proliferation was only enhanced in fibroblasts derived from cholesteatoma. This could be reversed by the treatment with a TLR4 antagonist. The cholesteatoma fibroblasts could be triggered by LPS to promote the epidermal differentiation of the stem cells, while no LPS treatment or LPS treatment without the presence of fibroblasts did not result in such a differentiation.

**Conclusion:**

We propose that cholesteatoma recurrence is based on TLR4 signalling imprinted in the cholesteatoma cells. It induces excessive inflammation of stem cells and fibroblasts, proliferation of perimatrix fibroblasts and the generation of epidermal cells from stem cells thru paracrine signalling by fibroblasts. Treatment of the operation site with a TLR4 antagonist might reduce the chance of cholesteatoma recurrence.

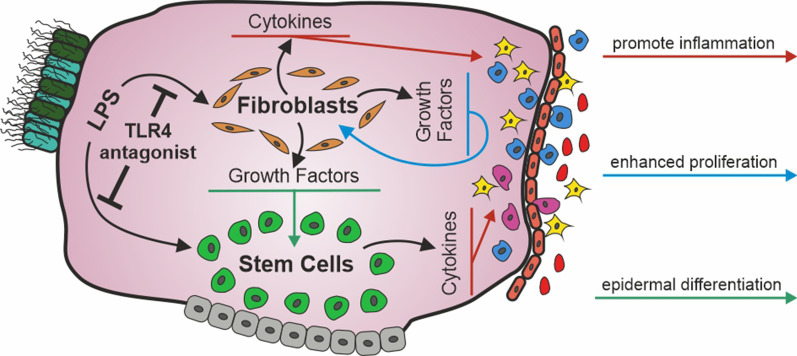

**Video Abstract**

## Background

The middle ear cholesteatoma is an expanding lesion of keratinizing epithelium in the middle ear leading to complications by eroding adjacent structures. The destruction of the ossicles may result in hearing loss, vestibular dysfunction and facial paralysis alongside with other intracranial complications may occur. This severe disease appears with a mean annual incidence of 9.2 per 100,000 among adult Caucasians [1]. Unfortunately, the only effective treatment of middle ear cholesteatoma is the surgical intervention.

On the histological level the middle ear cholesteatoma is characterized by epidermal cell hyperproliferation [2], differentiation and the accumulation of keratin debris [3]. Different theories for the pathogenesis exist [3, 4]. These theories are mainly based on either the relocation of keratinizing epithelium through the tympanic membrane into the middle ear or differentiation and hyperproliferation of epithelium due to inflammation. Interestingly, cholesteatoma rather mimics the inflammatory and proliferative phase of the wound-healing process without reaching maturation, e.g. displaying an abundant presence of fibronectin in cholesteatoma stroma [5] and proliferative stroma [6]. The most prominent pathogenic manifestation of a cholesteatoma, the hyperproliferative cholesteatoma epithelium, exhibits a high rate of Ki-67 [7] and proliferating cell nuclear antigen positive cells [8] compared to normal auditory skin. The enhanced proliferation is also manifested in hyperproliferative patterns of cytokeratin 16 and 19 in cholesteatoma epithelium [3]. The expression of cytokeratin 18 is known to be upregulated in cholesteatoma tissue compared to healthy auditory canal skin [9]. Furthermore cytokeratin 14, which is regularly expressed in mitotically active basal layer cells in normal skin and cholesteatoma [10], is expressed in cholesteatoma tissue in a higher extend compared to normal auditory canal skin [9]. The high state of inflammation in the cholesteatoma tissue is mainly caused by tissue damage and bacterial infection [11]. The gram-negative bacteria *Pseudomonas aeruginosa* and *Proteus mirabilis* are frequently found in cholesteatoma tissue, but also the gram-positive species *Staphylococcus aureus* represents a common pathogen [12]. It is particularly known that the Toll like receptor 4 (TLR4) is upregulated in acquired cholesteatoma [13, 14], which promotes a more severe progression of the disease by promoting inflammation and bone destruction [13]. Anyhow, the cause of this hyperproliferation is not fully understood, but it is known that TLR4 agonistic pathogen-associated molecular patterns (PAMPs) [15] as well as damage associated molecular patterns (DAMPs) inside the cholesteatoma tissue will activate the expression of different cytokines and growth factors provoking this proliferation [16]. In accordance to this Jovanovic et al. found that the most significantly differentially upregulated genes were linked to inflammation, epidermis development and keratinization [17]. In detail the expression of the cytokines, e.g. IL-1α [24] IL-1β and IL-6 [18], TNF-α [19], GM-CSF [20] and the chemokine IL-8 [21, 22], is elevated in cholesteatoma. Beyond this growth factors crucial for epidermal growth and wound healing, e.g. EGF [19, 22], KGF [23], Epiregulin [24], bFGF [25], TGF-β1 [26] and HGF [27], were upregulated as well in cholesteatoma tissue. The potent growth factor KGF was particularly associated with a high level of inflammation in cholesteatoma [28, 29] and correlated to its hyperproliferation [30].

Unfortunately, no curing medical treatment for cholesteatoma does exist, hence the surgical excision of cholesteatoma tissue appears to be the only effective treatment [31]. Unfortunately, the recurrence rate was estimated between 30% [32] and 12% [33]. Intriguingly, the recurrence rate is correlated to the inflammatory state of the tissue [34], hence physicians try to reduce the inflammation and secondary infections to prevent the recurrence by application of antibiotics and hydrocortisone. This study is designed to provide a deeper understanding of the process of cholesteatoma formation and recurrence by inflammation utilizing in vitro models. For this we utilized already established methods to isolate epidermal stem cells [14] and fibroblast [35] from cholesteatoma tissue. And demonstrated, that the cholesteatoma hyperproliferation and the differentiation of epidermal stem cells into keratinizing epithelium could be induced by inflammatory signaling. Most importantly, we found that anantagonistic blockage of TLR4 is sufficient to shut down the mechanisms underlying hyperproliferation and differentiation. We propose that the application of this antagonist offers a new medical approach to reduce the self-renewal capacity of cholesteatoma tissue remaining after surgery and hence the recurrence of cholesteatoma.

## Material method

### Source material and tissue preparation

Human cholesteatoma tissue (from posterior epitympanon) and auditory canal skin (from tympanomeatal flap) were obtained from patients after middle ear surgery at Klinikum Bielefeld Mitte (Bielefeld, Germany). The samples were obtained after fully informed and written consent prior to surgery according to local and international guidelines and all clinical investigations were ethically approved (Reg. no. 2235) and conducted according to the principles of the Declaration of Helsinki (1964) and local guidelines (Bezirksregierung Detmold/Münster). Immediately after removal the tissue samples were placed in Dulbecco’s Modified Eagle’s Medium (DMEM; Sigma Aldrich) on ice.

### Cell culture

The tissue was mechanically chopped with a scalpel and transferred into Collagenase class I and class II (0.375 U/ml in PBS with 3 mM CaCl_2_; SERVA Electrophoresis GmbH). After digestion the tissue samples were further mechanically dissociated by titration and pelleted by centrifugation (10 min., 300xg).

Stem cells isolated from cholesteatoma tissue (ME-CSCs) and from auditory canal skin (ACSCs) were cultivated in stem cell medium (SC-medium) consisting out of Dulbeccos’s Modified Eagle Medium: Nutrient Mixture F12 (DMEM/F12; Sigma Aldrich) containing 200 mM L-Glutamin (Sigma Aldrich), human epidermal growth factor (EGF, 20 ng/mL; PeproTech), basic fibroblast growth factor (bFGF, 40 ng/mL; PeproTech), B-27 Supplement (3%; Life Technologies), amphotericin B (25 µg/mL; Sigma Aldrich), penicillin and streptomycin (10 U/mL; Sigma Aldrich). For initial expansion of stem cells 10% blood plasma was added to the medium. To further expand stem cells ME-CSCs and ACSCs were deliberated from the fibrin matrix by Collagenase class I and class II (0.375 U/ml in PBS with 3 mM CaCl_2_; SERVA Electrophoresis GmbH) and cultured in low adhesion 25 mm^2^-tissue culture flasks (Sarstedt) as free-floating spheres in SC-medium supplemented with heparin (2 µg/mL; Sigma Aldrich). To passage spheres the cells aggregates were dissociated via Accutase (PAA Laboratories GmbH) for 10 min. at 37 °C.

For Fibroblasts isolation, the cells derived from the digested tissue were cultivated in FB-medium consisting out of DMEM containing 200 mM L-Glutamin (Sigma Aldrich), amphotericin B (25 µg/mL; Sigma Aldrich), fetal calf serum (FCS, 10%; Capricorn Scientific) penicillin and streptomycin (10 U/mL; Sigma Aldrich). The FCS was assayed for LPS concentration by the distributor. The LPS concentration for the used batch number was 3.68 ng/ml, resulting in a LPS concentration of 0.37 ng/ml in the FB-medium. The isolated fibroblasts from cholesteatoma tissue and auditory canal skin were termed ME-CFs and ACFs, respectively. Passaging was executed by trypsination when cells reached 80 – 90% confluence.

### Cell stimulation experiments

Cells were seeded in 6-well plates (CytoOne®; STARLAB GmbH) with a density of 5 × 10^4^ cells/well. After overnight (o/n) incubation in FB-medium cells were treated with 100 ng/mL LPS (rough strain from Salmonella enterica Re 595; Sigma Aldrich). Alternatively, cells were stimulated simultaneously with LPS as a TLR4 agonist (100 ng/mL; Sigma Aldrich) and 10.000 ng/mL LPS from Rhodobacter sphaeroides (LPS-RS, InvivoGen) as a TLR4 antagonist. The control samples were treated with FB-medium. After four hours of stimulation, medium was removed and cells were gently washed with 1 × PBS and processed for RNA isolation.

### RT-qPCR

RNA isolation was performed using the innuPREP RNA mini Kit (Analytik Jena) according to the manufacturer’s protocol. The RNA concentration and purity was measured by NanoPhotometer® classic (IMPLEN). Afterwards the RevertAid First Strand cDNA Synthesis Kit (ThermoFisher Scientific) was utilized for reverse transcription.

For quantitative real-time polymerase chain reaction (qPCR) the Luna® Universal qPCR Master Mix (NEB) was used according to manufacturer’s guidelines. The reactions were executed in technical triplicates for each primer pair (cf. Table 1). In addition, a no template control was performed. The MIC qPCR cycler (Bio Molecular Systems) was used for product detection. Glyceraldehyde 3-phosphate dehydrogenase (GAPDH) expression was utilized as reference gen and the according relative expression was depicted or this expression was normalized to 100%. Prism (GraphPad Software) was applied for graphics and statistical analysis.

**Table 1:** Used primer sequences for qPCR

Primer	Sequence (5′ → 3′)	Size of product (bp)
bFGF	CTGGCTATGAAGGAAGATGGA TGCCCAGTTCGTTTCAGTG	149
Cytokeratin 14	CTCTAGTGCTGTCACCCAGTT CACAGACACCACGTAGAAGCA	120
Cytokeratin 16	AAAGCATCCCTGGAGAACAGC CCTCCACACTGCCAATCAGTC	91
Cytokeratin 18	GACCGCCTGGCCTCTTAC ACCTGGGGTCCCTTCTTCTC	104
Cytokeratin 19	GAATCGCAGCTTCTGAGACCA CTGGCGATAGCTGTAGGAAGT	74
EGF	GCTGTGTCATTGGATGTGCT TCACCAAAAAGGGACATTGC	161
EREG	TATCACAGTCGTCGGTTCCA AACTCTGGATCCCCTGAGGTA	108
GAPDH	CTGCACCACCAACTGCTTAG GTCTTCTGGGTGGCAGTGAT	108
GM-CSF	TCCTGTGCAACCCAGATTATCA TCATCTGGCCGGTCTCACTC	116
HGF	TGACACGTAGGCTGGAACTG AGTTTGGTGGTCTCCATTGCT	74
IGF-2	ACGTTCACTCTGTCTCTCCCA CGGGCCAGATGTTGTACTTT	109
IL-1α	TGCCTGAGATACCCAAACC GCCAAGCACACCCAGTAGTC	145
IL-1β	TGTACCTGTCCTGCGTGTTGAAAG CTGGGCAGACTCAAATTCCAGCTT	149
IL-6	GCAAAGAGGCACTGGCAGAAAACA TTCTGCAGGAACTGGATCAGGACT	226
IL-8	TCTCTTGGCAGCCTTCCTGATTTC AGTTTTCCTTGGGGTCCAGACAGA	227
KGF	CAGTGGCAGTTGGAATTGTG CCTCCGTTGTGTGTCCATTT	178
Ki-67	AGTGCTGATGGTTTACAGGGG AGACTCCACGTCTCTTCCCT	150
TGF-β1	GAGCCCTGGACACCAACTAT GTCCAGGCTCCAAATGTAGG	167
TLR4	CACAGACTTGCGGGTTCTACATCA TGGACTTCTAAACCAGCCAGACCT	192
TNFα	AAGCCCTGGTATGAGCCCATCTAT AGGGCAATGATCCCAAAGTAGACC	137

### MTT assay—measurement of metabolic activity

For measurement of cell metabolic activity, cells were seeded in 12-well plates (CytoOne® Multiple Well Plates; STARLAB GmbH) with a density of 1.7 × 10^3^ cells/well in FB-medium. After o/n incubation in FB-medium (37 °C, 5% CO_2_) cells were stimulated with 100 ng/mL LPS (Sigma Aldrich), while the control remained untreated. For day 0, the metabolic activity was determined from untreated cells. Moreover, the measurement of metabolic activity of the cells was done at three further time points as indicated in the graph. Every day half of the medium was removed and replaced with the corresponding medium. For measurement of metabolic activity the medium was removed and replaced with FB-medium without phenol red containing 0.5 mg/mL 3-(4,5-Dimethyl-2-thiazolyl)-2,5-diphenyl-2H-tetrazoliumbromid (MTT; Sigma Aldrich) and incubated for 2 h (37 °C, 5% CO_2_) with or without 100 ng/mL LPS. To dissolve arose formazan crystals dimethylsulfoxid was added preceding a 10 min incubation (37 °C, 5% CO_2_). The cell debris in the solution was removed by centrifugation (3 min.; 8000 g). Afterwards the quantification of metabolic activity was measured via NanoPhotometer® classic (IMPLEN) at 570 nm and 690 nm. The absorbance of 690 nm was subtracted from the absorbance of 570 nm. The measurement of metabolic activity of stimulated and control cells were made in technical triplicates for each time point. Prism (GraphPad Software) was used for analysis and graphics depiction.

### Proliferation assay—measurement of doubling time

Cells were seeded in 6-well plates (CytoOne®, STARLAB GmbH) having a density of 5 × 10^4^ cells/well. After o/n incubation in FB-medium cells were stimulated with 100 ng/mL LPS (Sigma Aldrich) or left untreated. Every day half of the medium was exchanged with the corresponding medium. At three further time points, marked in the graph, the cell number of treated and untreated cells were determined. Cells were harvested through trypsination, pelleted, resuspended in 100 µl of FB-medium and counted with a Neubauer chamber. For graphics and statistical analysis Prism (GraphPad Software) was employed.

### In vitro model of cholesteatoma progression

To simulate paracrine stimulation of ME-CSCs by ME-CFs during cholesteatoma progression we used an indirect co-culture model. The ME-CSCs were seeded in SC-medium with a density of 10^4^ cells/cm^2^ in cell culture inserts (12-well Millicell®, Millipore) coated with poly-d-lysine. Simultaneously, ME-CFs were seeded in SC-medium with a density of 2 × 10^4^cells/well in 12-well plates (STARLAB GmbH)coated with poly-d-lysine. After o/n incubation the ME-CSCs were transferred to empty 12-wells or wells containing the ME-CFs. Subsequently, the insert as well as the 12-wells were filled with 1 ml of fresh SC-medium either with or without 100 ng/ml LPS (Sigma Aldrich). The medium in the 12-wells was changed every 2–3 days while the medium in the insert was left unchanged. After two weeks of cultivation the ME-CSCs were either lysed and further processed for RT-qPCR or prepared for Immunocytochemistry.

### Immunocytochemistry

For immunocytochemical staining of co-cultivated ME-CSC the membrane of the cell culture insert cells was removed from its retainer. Fixation of cells was done with 4% paraformaldehyde (PFA; Sigma Aldrich; 20 min., 4 °C). This step was followed by washing with 1 × PBS (3 × 5 min.) at room temperature (RT). Afterwards, cells were permeabilized and blocked with a solution of 0.02% TritonX-100 (AppliChem, Darmstadt) and 1% BSA in 1 × PBS (30 min., RT). Subsequently the primary antibody (cf. Table 2) was applied o/n at 4 °C. Subsequently, cell culture membranes were washed with 1 × PBS (3 × 5 min). Following that, the secondary fluorochrome-conjugated antibody (cf. Table 2) was applied (1 h, RT). Thereafter, three more washing steps with 1 × PBS (5 min.) were followed by nuclear counter staining with 4′,6-Diamidin-2-phenylindol (DAPI, 1 µg/ml; Sigma Aldrich) (15 min, RT). After two more washing steps with 1 × PBS (5 min.) and one washing step with H_2_O (10 s) the membranes were embedded in mowiol and gently pressed down during curing to improve their evenness. Samples were imaged via confocal laser scanning microscopy (LSM 710; Carl Zeiss).

**Table 2:** Antibodies used for immunocytochemistry

	Species	Number	Manufacturer	Dilution	Incubation
*Primary antibody*					
Cytokeratin 14	Rabbit	PA5-28,002	Invitrogen	1:200	4 °C, o/n
Cytokeratin 16	Mouse	sc-377224	Santa Cruz	1:100	4 °C, o/n
Cytokeratin 18	Mouse	SC-32329	Santa Cruz	1:500	4 °C, o/n
Cytokeratin 19	Rabbit	NBP1-78,278	Novus Biologicals	1:100	4 °C, o/n
*Secondary antibody*					
Anti-rabbit Alexa 488	Goat	A32731	Invitrogen Molecular Probes	1:300	RT, 1 h
Anti-mouse Alexa 555	Goat	A21422	Invitrogen Molecular Probes	1:300	RT, 1 h

## Results

### The isolated cells differ in expression of some inflammatory mediators, growth factors and the expression von TLR4

**Fig. 1:**
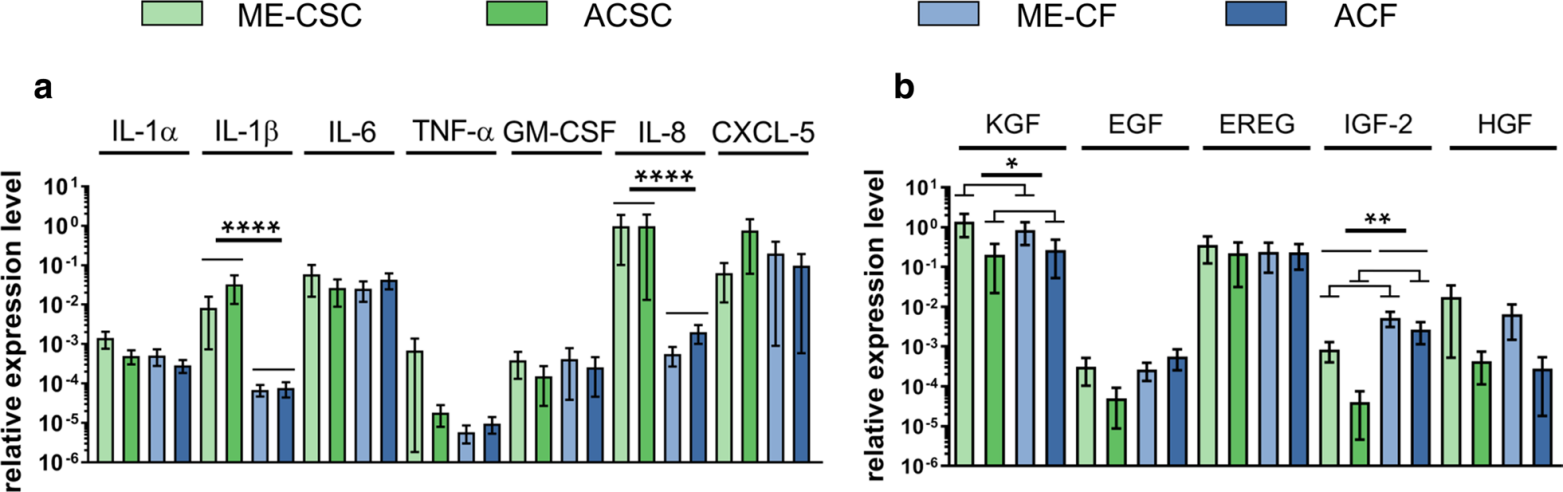
Basic expression level for the four cells types under standard culture conditions derived from RT-qPCR data (n = 3). **a** The relative expression level of cytokines and chemokines in the investigated cell types. Except for two highly significantly upregulated markers in stem cells (IL-1β and IL-8), these signalling molecules exhibit no significant tendency between samples or cell types. **b** The relative expression of growth factors for all cell types. Again a slight but significant upregulation in fibroblasts can be detected for IGF-2. Besides that, cholesteatoma cells exhibit a significantly higher expression of KGF and IGF-2. (Depicted: mean and standard deviation of mean; *≤ 0.05, **≤ 0.01, ****≤ 0.0001, on tailed ration paired t-test, 95% confidence interval upon passed Shapiro–Wilk normality test)

Using two different cell culture techniques, we were where able to derive two different subpopulations of cells from each of these two tissues. One type were fibroblasts derived from cholesteatoma and auditory canal skin (ME-CF and ACF respectively) the other one were stem cells of epidermal origin (ME-CSCs and ACSCs respectively) already described in [14, 36]. Targets responsible for inflammation and wound healing were investigated by RT-qPCR in all cell types under standard culture conditions (Fig. 1). The chosen targets were different cytokines, chemokines and growth factors known to be overexpressed in cholesteatoma tissue and/or related to cholesteatoma development and its pathological exaggerated inflammatory niche. The relative expression levels for one specific cell type were distributed between the different donors over 3 orders of magnitude (OM) in case of TNF-α or IL-8 or just slightly different e.g. in the case if IL-1 α. Despite this huge biological variance, a significantly higher expression in ME-CSCs and ME-CFs derived from cholesteatoma tissue could be observed for the growth factors KGF and IGF-2 (*p* ≤ 0.05, *p* ≤ 0.01 respectively). A similar trend could be assumed for the growth factor HGF and the cytokine IL-1α even though it did not reach the level of statistical significance (*p* = 0.07 in both cases). More pronounced was the tissue-independent trend to higher expression of the inflammatory mediators IL-1β and IL-8 in stem cells (around 250 fold and 700 fold, respectively, *p* ≤ 0.0001) and the growth factor IGF-2 in fibroblasts (between 6 and 60 fold). Since the TLR4 is known to play a fundamental role in the inflammatory environment of the cholesteatoma, we furthermore investigated its expression level in the cells of three different donors upon cultivation in FB-medium, as this medium was used in the following LPS stimulation experiments presented below.

**Fig. 2:**
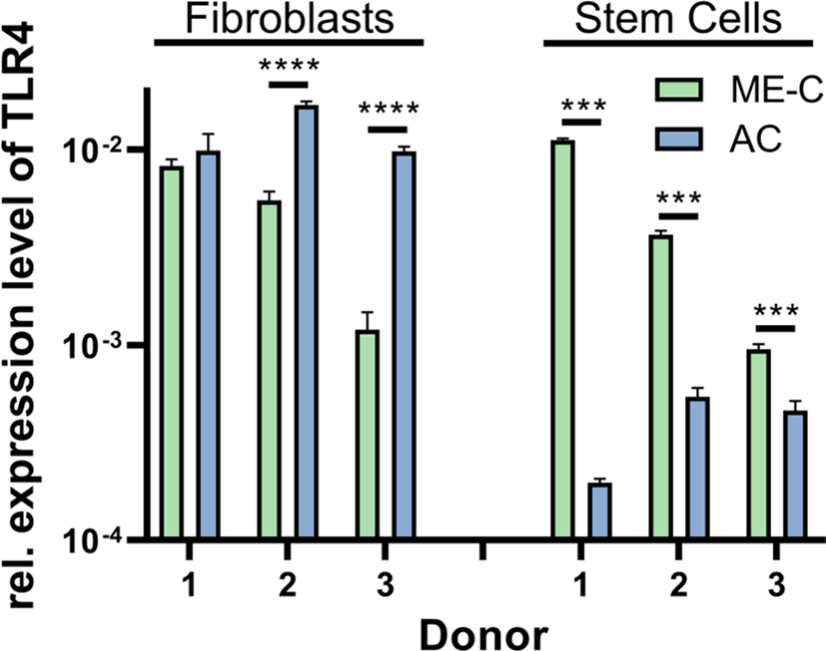
The relative expression level of TLR4 in the different cell types investigated by RT-qPCR (n = 3). The expression level in fibroblasts (left) showed variation over one OM with a tendency to higher expression in cells derived from auditory canal skin. The stem cells exhibited a higher variation over nearly two OM and showed a clear trend to higher TLR4 expression in ME-CSCs (depicted: mean and standard deviation of mean; unpaired one tailed t-test with Welch correction, 95% confidence interval, upon passed Shapiro–Wilk normality test; ***≤ 0.001, ****≤ 0.0001)

For cholesteatoma tissue, the ME-CSCs and ME-CFs showed a similar distribution of TLR4 expression between 1 and 0.1% relative to GAPDH (Fig. 2). In regards to the tissue of origin the obtained data showed an opposed effect for fibroblasts and stem cells. Interestingly, the TLR4 expression tended to be lower in fibroblasts and higher in stem cells derived from cholesteatoma compared to the same cell type from auditory canal skin. In numbers, the reduced expression in ME-CFs compared to ACFs range from rather insignificant 87% down to highly significant 15% (*p* ≤ 0.0001), while all ME-CSCs showed a significant upregulation between 55 and twofold compared to ACSCs (*p* ≤ 0.001).

To investigate the reaction towards a bacterial infection in the four different cell types, we stimulated the cells with LPS and measured the transcription of different inflammatory mediators and growth factors. The stimulation was executed with 100 ng/ml, whereas the control contained only 0.37 ng/ml. We consider this as an LPS-free control since even at 1 ng/ml no significant stimulation could be detected (Additional file 1: Fig. S1).

**Fig. 3:**
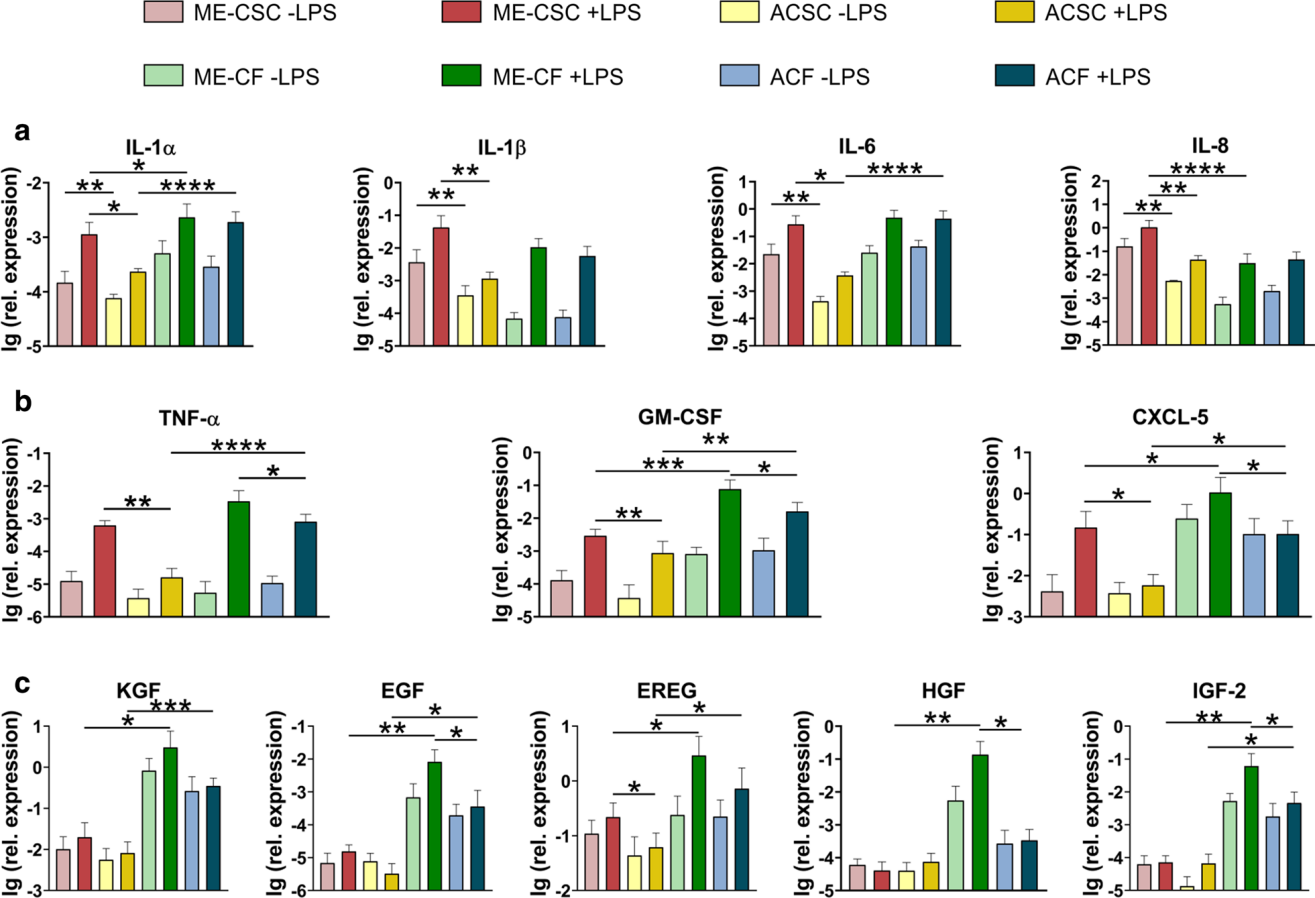
The relative expression level of transcripts in stem cells and fibroblasts derived from the two different tissues with and without stimulation with LPS (n = 3). **a** Transcripts of the interleukin family (IL-1α, IL-1β, IL-6, IL-8). All transcripts are significantly increased in ME-CSCs compared to ACSCs with or without stimulation with LPS. Additionally, the expression was heavily increased in stimulated ME-CFs in relation to ME-CSCs (IL-1α) but massively decreased in ME-CFs relative to ME-CSCs (IL-8). **b** Upon stimulation with LPS, three other modulators of Immune response (TNF-a, GM-CSF and CXCL-5) exhibited an significant increase in ME-CSCs and ME-CFs compared to ACSCs and ACFs, respectively. Furthermore, the transcription of all transcripts was elevated for ME-CFs in relation to ME-CSCs in the case of GMCSF and CXCL-5. **c** Intriguingly, the expression of all investigated growth factors (KGF, EGF, EREG, IGF-2 and HGF) was significantly increased in ME-CFs and ACFs (with exception of HGF). The expression of EREG was elevated in ME-CSCs compared to ACSCs while EGF, HGF and IGF-2 were increased in ME-CFs in relation to ACFs. (Depicted: mean and standard deviation; statistics between cell types: one tailed Wilcoxon matched-pairs signed rank test, statistics between tissue of origin: one tailed Mann–Whitney-U both with 95% confidence interval,*≤ 0.05, **≤ 0.01, ***≤ 0.001, ****≤ 0.0001)

We grouped these transcripts into three classes, according to their characteristic reaction in the cell types upon stimulation. The first group comprised targets from the interleukin family (Fig. 3a). A tissue specific difference with and without LPS stimulation was detected in stem cells while in fibroblasts no such effect could be observed. The cell type specific difference between fibroblasts and stem cells derived from cholesteatoma tissue was rather marginal, showing relations between 200 and 25% which reached statistical significance infrequently. An exception was IL-8 exhibiting a downregulation to about 3% in ME-CFs (*p* ≤ 0.0001). Since ACSFs also expressed low levels of IL-8, the high IL-8 expression was specific for ME-CSCs. Another interesting trend could be observed: the expression of IL-6 was heavily elevated for ME-CSCs. A pattern of fibroblast-specific upregulation was measured for IL-1α. Since ME-CSCs did not show comparable expression levels as ME-CFs upon stimulation with LPS, IL-1α seems to be a rather fibroblast-specific target.

The second group of targets includes different inflammatory mediators, which reacted with a higher sensitivity upon LPS stimulation in all cell types derived from cholesteatoma tissue (Fig. 3b). The expression levels of different markers in ACSCs in relation to ME-CSCs lays at 2.5% (TNF- α, *p* ≤ 0.01, 3.5% (CXCL-5, *p* ≤ 0.05) and 30% (GM-CSF, *p* ≤ 0.01). This tissue specific difference is also distinctive for ACSFs, for which the expression levels were detected at around 2.2% (TNF-α, GM-CSF) and 10% (CXCL-5) of those values measured for ME-CFs (*p* ≤ 0.05). In this group, also the expression with and without LPS stimulation was much higher in fibroblasts independent of the tissue of origin. In average, the expression levels in stem cells reached 2–20% (TNF-a), 4–5% (GM-CSF) and 5–14% (CXCL-5) of the levels detected in fibroblasts (*p* ≤ 0.01), making all these targets specific for fibroblasts. The last group comprises all growth factors investigated in this study (Fig. 3c). The growth factors are characterised by a huge upregulation in expression in ME-CFs and also in ACFs, even though to a much lesser extent. In detail, the expression was elevated for ME-CFs and ACFs compared to their corresponding stem cells 160 fold and 30 fold (KGF) (*p* ≤ 0.01 and *p* ≤ 0.0001), 530 fold and 110 fold (EGF) (*p* ≤ 0.01and *p* ≤ 0.05), 13 fold and 11 fold (EREG) (*p* ≤ 0.05), 340 fold and fourfold (HGF) (*p* ≤ 0.01 and ns), and 860 fold and 75 fold (IGF-2) (*p* ≤ 0.01and *p* ≤ 0.05), respectively. In this group, only a random tissue specific response to the LPS stimuli could be detected. This response was rather weak for EREG in stem cells (3.5 fold, *p* ≤ 0.05) and more pronounced in fibroblasts for IGF-2 (13 fold), EGF (23 fold), and especially HGF (450 fold) (*p* ≤ 0.05). Interestingly, HGF is the only target which seems to be specific in a tissue and cell type specific manner for ME-CFs.

Since we detected an abnormal expression of inflammatory mediators and growth factors for cells derived from cholesteatoma tissue upon stimulation with LPS, we decided to measure the effect of LPS on the metabolic activity and proliferative behaviour of ME-CSCs and ME-CFs.

**Fig. 4:**
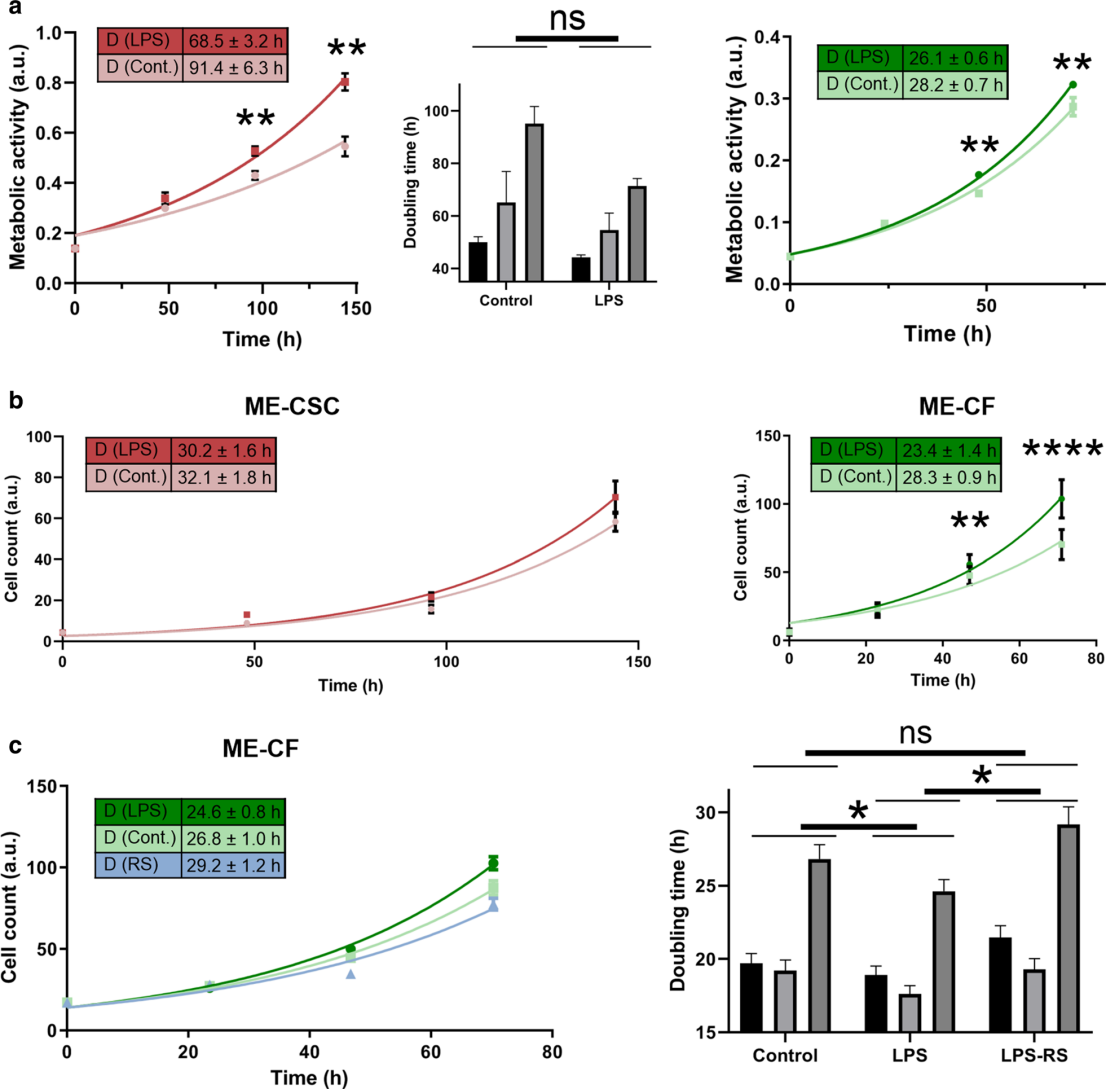
Metabolic and proliferative behaviour of cells derived from cholesteatoma tissue stimulated with LPS. **a** A MTT assay of ME-CSCs and ME-CFs with or without stimulation with LPS. Some ME-CSCs showed a significantly higher metabolic activity. A broader investigation (n = 3) could not verify the significance of this enhanced metabolism. In ME-CFs the metabolic activity was already enhanced after three days of cultivation. **b** Proliferation assay of ME-CSCs and ME-CFs derived from the same donor. The ME-CSCs showed only a small and insignificant enhancement in proliferation while the ME-CF exhibited a drastic change in mitotic activity upon LPS stimulation. **c** Proliferation assay executed with the same ME-CFs as shown in (**b**) with and without LPS stimulation and with the LPS quenched by the antagonists LPS-RS (left). A clear reduction in doubling time is detectable for the LPS-treated and even a bit for the control population. Biological triplicates of this experiment (right) demonstrated, that this effect is statistically significant (depicted: doubling time and standard deviation derived from exponential curve fit; one tailed paired (for bar diagrams) and one tailed non paired t-test (for data points in the xy-graphs)with 95% confidence interval upon passed Shapiro–Wilk normality test, ns ≥ 0.05, *≤ 0.05, **≤ 0.01,****≤ 0.0001)

To investigate the biological effect of the increased production of inflammatory mediators and growth factors on the two different cell types derived from cholesteatoma tissue, we measured the metabolic activity upon long-term exposure of ME-CSCs and ME-CFs to LPS. For ME-CSCs we could detect an increase in metabolic activity for one of the investigated three donors after 6 days (Fig. 4a). From an exponential curve fit, a reduction in doubling time for the metabolic activity from 91.4 ± 6.3 down to 68.5 ± 3.2 days for ME-CSCs was derived (≤ 0.01). Repetition of this experiment resulted in no statistical significance of this effect. For ME-CFs, even after only two days of cultivation a significant change in metabolic activity was observed (from 28.2 ± 0.7 down to 26.1 ± 06, *p* ≤ 0.01).

To investigate the mechanisms underlying the increased metabolic activity, we executed proliferation assays using cells of the same donors as investigated by the MTT assay (Fig. 4b). The examined ME-CSCs exhibited only a slight and insignificantly increased mitotic activity even after 6 days of stimulation with LPS. The exponential fit of the growth data resulted in a similar doubling time of 32.1 ± 1.8 h without LPS and 30.2 ± 1.6 with stimulation by LPS. When executing the same experiment with ACFs derived from the same patients no such LPS-dependent stimulation of proliferation could be detected (Additional file 2: Fig. S2). In contrast to that, the stimulation of ME-CFs with LPS lead to a significant increase in proliferation, with doubling times of 28.3 ± 0.9 h and only 23.4 ± 1.4 h without stimulation (*p* ≤ 0.0001), detectable even 4 days after the addition of LPS into the medium.

To rescue the LPS-treated phenotype of ME-CSCs owning an enhanced proliferation, we repeated the proliferation assay with ME-CFs derived from three different donors with the application of the TLR4 antagonist LPS-RS, which was added into the LPS-supplemented medium (Fig. 4c). Again a significant increase in proliferation of ME-CFs was detected upon treatment with LPS (*p* ≤ 0.01).

By comparing the derived doubling times, we were able to show that LPS-RS is able to reduce the proliferation of ME-CFs cultured with LPS. To be more specific, the addition of LPS-RS into the medium returned the mitotic activity to the one observed at standard culture conditions. The downregulation of transcripts responsible for fibroblast proliferation upon blockage of the TLR4 receptor by LPS-RS (Additional file 3: Fig. S3) even below the control level, illustrates one of the sources for this rescue effect.

**Fig. 5:**
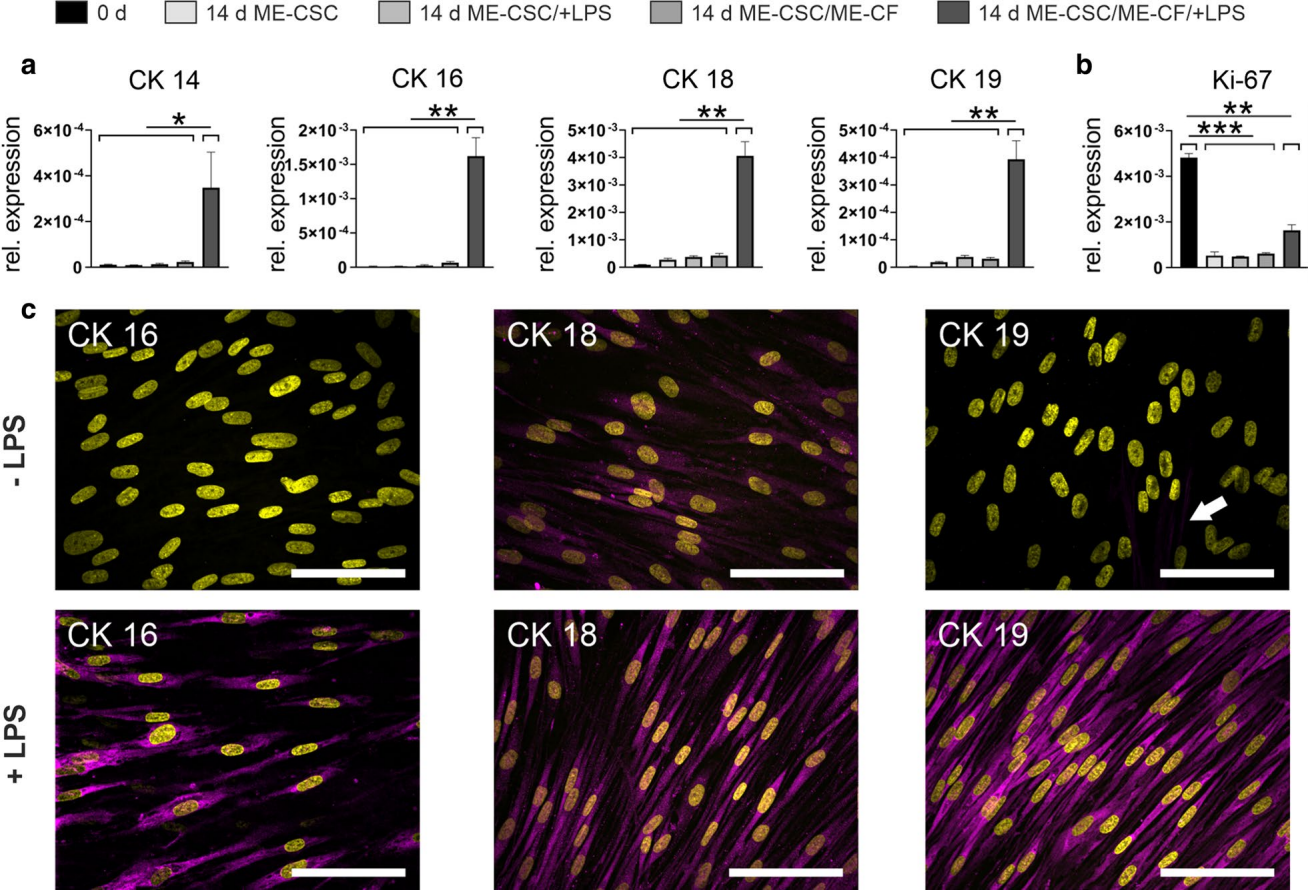
Promotion of epidermal differentiation of ME-CSCs in a co-culture in vitro model of cholesteatoma recurence. **a** The expression of different cytokeratins in ME-CSCs in the co-culture system after 14 days with or without treatment with LPS and with or without simultaneous co-culture with ME-CFs. Additionally the control of the untreated cells under standard culture conditions is shown. Only the co-culture treated with LPS showed a highly significant increase in the expression of these cytokeratins. **b** The expression of Ki-67 as marker for proliferation of the same samples depicted in (**a**). The mitotic activity is reduced for all samples relative to the control but the LPS treated co-culture shows a slight increase in Ki-67 expression relative to the other samples cultured for 14 days. **c** LSM imaging of ME-CSCs co-cultivated with fibroblasts in medium containing LPS or control medium. The immunofluorescence staining of cytokeratin 16, 19 reveals that these two cytokeratins are homogenously induced in ME-CSCs by stimulation of the fibroblasts. Cytokeratin 19 is also sparsely expressed in control culture (arrow). The expression of cytokeratin 18 is likewise induced in the stimulated culture, but also to a lesser extent in the control medium; (depicted: mean standard deviation; one tailed non paired t-test with 95% confidence interval upon passed Shapiro–Wilk normality test, *≤ 0.05, **≤ 0.01, ***≤ 0.001)

To examine if the growth factors expressed by the ME-CFs exert a differentiating effect on the ME-CSCs we designed an indirect cell culture insert-based co-culture model of cholesteatoma progression and  recurence. In this model, the fibroblasts and stem cells were co-cultivated in medium containing LPS. As controls served the same setup without LPS supplementation, ME-CSCs cultivated without the presence of ME-CFs (with or without LPS), and ME-CSCs cultivated under standard cell culture conditions. RT-qPCR analysis showed a remarkable and significant upregulation of cytokeratins in ME-CSCs after 14 days of co-culture stimulated with LPS (Fig. 5a). The expression of cytokeratin 14 was upregulated 15-fold compared to ME-CSCs co-cultured without LPS and 30-fold relative to culture conditions without LPS and co-cultivation (*p* ≤ 0.05). For cytokeratin 16, cytokeratin 18 and cytokeratin 19 the corresponding fold changes were 25-fold and 210-fold, ninefold and 45-fold, and 12 fold and 150 fold, respectively (*p* ≤ 0.01). In the co-culture with LPS the relative expression level was highest for cytokeratin 16 and cytokeratin 18 with relative expression compared to the house keeping gene of 1.6 ‰ and 4 ‰, respectively, and lower for cytokeratin 14 and cytokeratin 19 with both showing a relative expression of only 0.3‰. The expression of Ki-67 was significantly reduced in all samples cultivated over 14 days from 3 to tenfold (*p* ≤ 0.01, except LPS treated co-culture showing *p* ≤ 0.05). Intriguingly, the expression in the co-culture system comprising LPS was shown to be elevated compared to the other ME-CSCs samples by a factor of 3. In ME-CSCs co-cultured with LPS treated ME-CFs, this decrease of proliferation was less pronounced. The subsequent immunofluorescence for these cytokeratins revealed, that cytokeratins 16 and 19 are heavily upregulated in ME-CSCs co-cultivated with LPS-stimulated fibroblasts (Fig. 5c). Even though cytokeratin 19 was also irregularly expressed in ME-CSCs cultivated with non-stimulated ME-CFs, but this was a rare observation. Cytokeratin 18 was also homogenously induced in the control cells on protein level, but to a smaller extent.

## Discussion

In this study, weinvestigated the mechanisms underlying the recurrence of cholesteatoma tissue. We demonstrated, that the long term repopulation capacity of stem cells in combination with autocrine and paracrine mechanisms involving fibroblasts is able to promote this recurrence upon stimulation with TLR4 agonists.

While investigating the expression of targets related to cholesteatoma disease under standard culture conditions (Fig. 1), we detected that the expression of inflammatory mediators IL-1β and IL-8 was massively enhanced in stem cells compared to fibroblasts, while IL-1α and TNF-α exhibited this effect in a less pronounced manner. For the growth factor IGF-2, fibroblasts showed higher levels compared to stem cells. When comparing the cells derived from cholesteatoma tissue with the cells from auditory canal skin, we could observe a significant upregulation of the two growth factors KGF and IGF-2 and a similar trend for IL-1α and HGF. This enhanced expression in vitro fits to the higher expression of KGF [23], IL-1α [37] and HGF [27] detected in vivo in cholesteatoma tissue when compared to auditory canal skin. In fact, IL-1α expression could be localized in cells of the perimatrix tissue [37]. Furthermore, the enhanced expression of KGF was already detected by Raffa et al. [38] when ME-CFs were compared to non-auricular skin.

It is known that LPS plays an important role in the progression of cholesteatoma, e.g. by directly triggering cholesteatoma keratinocyte proliferation [39]. Of course, LPS helps to generate the destructive proinflammatory environment in cholesteatoma tissue by stimulating the TLR4 in various cell types present in cholesteatoma tissue. Considering this, it is of particular interest, that the TLR4 is upregulated in acquired cholesteatoma [13, 14, 40], and especially strongly expressed its perimatrix [41]. We determined a similar TLR4 expression in the two cell types derived from the perimatrix (ME-CSCs and ME-CFs) under the culture conditions applied during LPS stimulation (Fig. 2). In the subsequent LPS stimulation of the four different cell types we applied a concentration of 100 ng/ml LPS which conforms to the LPS concentration of 85 ± 6.5 ng/ml found in purulent inflamed cholesteatoma tissue by Peek et al. [42]. Titration of LPS between 1 µg/ml and 0 ng/ml demonstrated that there was no significant difference between the stimulation with 1 ng/ml and 0 ng/ml (Additional file 1: Fig. S1). Thus we concluded that our LPS free FB-medium, known to contain 0.37 ng/ml LPS, was suitable to simulate the inflammatory state in non-purulent cholesteatoma tissue containing 0.003 ± 0.5 ng/ml LPS [42]. Hence the utilized LPS stimulation represents the LPS concentration in uninflamed and inflamed cholesteatoma tissue. Based on the response of the different cell types we differentiated the investigated targets into three groups. The first group comprised the cytokines IL-1α, IL-1β, IL-6 and the chemokine IL-8 in all these targets the ME-CSCs reacted stronger than ACSCs to high and low LPS concentrations. We decided to term this effect tissue memory effect. No such tissue memory effect was detectable in ME-CFs. Interestingly, the chemoattractant IL-8 was expressed nearly 2 OM more in ME-CSCs compared to ME-CFs. Hence we suggest that ME-CSCs contribute significantly to the high expression of IL-8 observed in cholesteatoma tissue [9, 30].

**Fig. 6:**
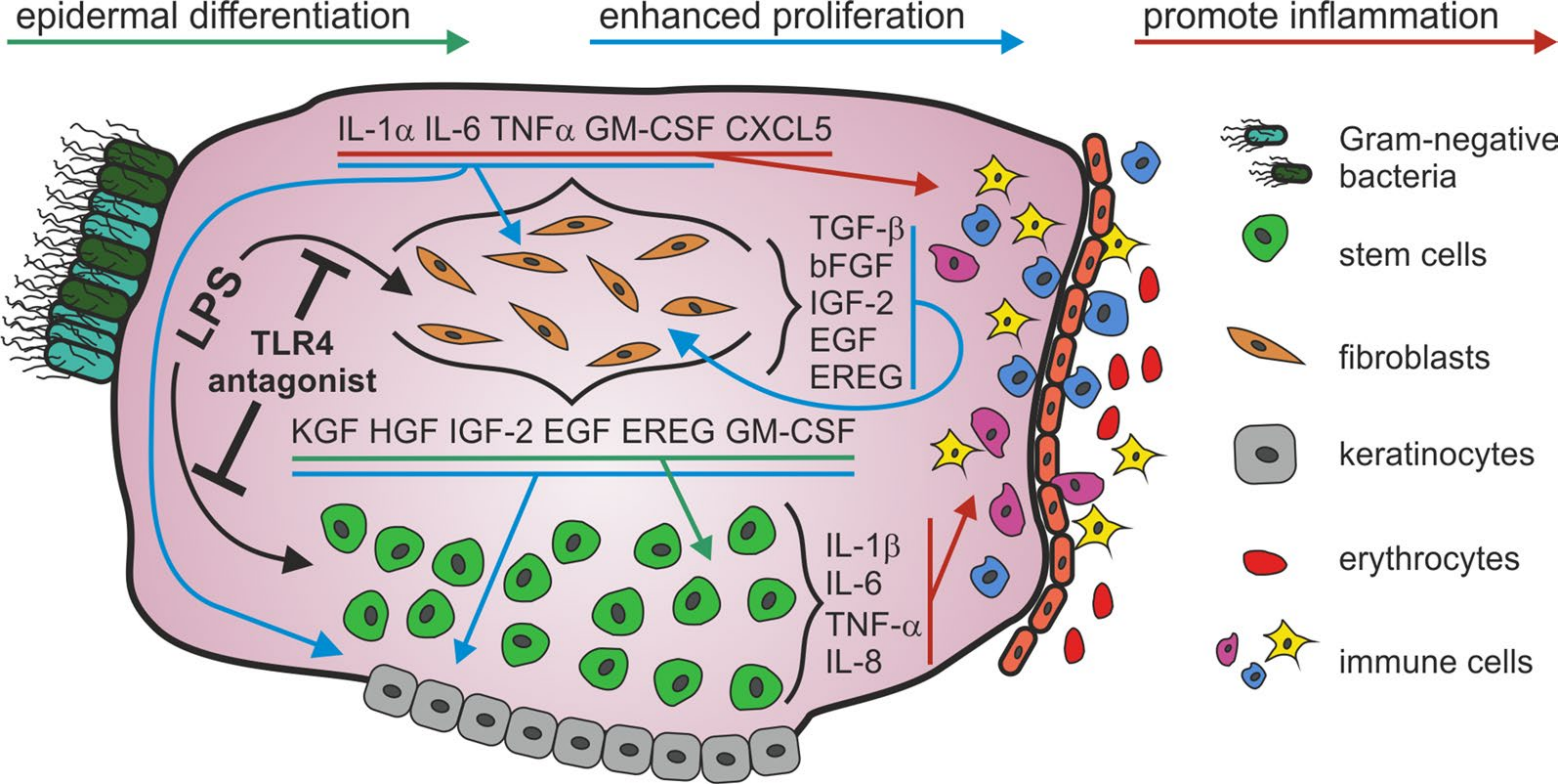
Primary paracrine and autocrine signalling contributes to cholesteatoma pathogenesis upon activation of the TLR4 pathway

The second group was characterized by a tissue memory effect in both cell types at high LPS concentrations and included all other inflammatory mediators (TNF-α, GM-CSF and CXCL-5). In this group the expression of GM-CSF and CXCL-5 was almost two and one OM higher respectively in ME-CFs compared to ME-CSCs. Indeed it is known that cholesteatoma-associated fibroblasts, which are unable to turn off their inflammatory program, play a critical role in the switch from acute to chronic inflammation [43]. We assume that the tissue memory effect observed for inflammatory mediators in cells residing in cholesteatoma tissue reflects an epigenetic adaptation to the chronic inflammation in cholesteatoma disease. By this effect the ME-CSCs and ME-CFs induce the exaggerated recruitment of immune cells (Fig. 6). While the cause for the tissue memory effect of stem cell could be partly explained by the enhanced expression of TLR4, this derivation cannot be applied for ME-CFs, which exhibit a downregulation of TLR4 compared to control tissue (cf. Figure 2). Anyhow, in case of ME-CSCs we also could detect a hypersensitive NF-κB pathway downstream of the TLR4 receptor [44]. Indeed, stem cells represent a good storage medium for epigenetic memories, since they hand their inflammatory experiences down to their offspring due to their long-term repopulating capacity. In accordance to this, an inflammatory memory could be demonstrated in epidermal stem cells of the skin [45].

The third category comprised all investigated growth factors (KGF, EGF, EREG, HGF and IGF-2) this group of targets was characterized by their significantly higher expression in ME-CFs compared to ME-CSCs. This difference reached from “only” around one OM for EREG over two OM in the case of KGF up to extreme expression differences of three OM for EGF, HGF and IGF-2. Hence we suggest that ME-CFs are the main source of growth factors in cholesteatoma perimatrix in a similar fashion as during normal wound healing [46]. The tissue memory effect was sporadic in ME-CSCs, while ME-CFs did show a significant tissue memory effect for the growth factors EGF, HGF and IGF-2 and a certain trend for KGF and EREG.

Upon verification of the enhanced susceptibility of ME-CSCs and ME-CFs to LPS stimulus, we investigated if such stimulus might contribute to cholesteatoma pathogenesis. Since cholesteatoma tissue is known to be hyperproliferative, we firstly investigated the ability of the secreted inflammatory mediators and growth factors to promote cell proliferation. Due to its convenience we initially applied a MTT assay to detect differences between LPS treated and untreated cells derived from cholesteatoma tissue. ME-CSCs showed only a slight but not significantly enhanced metabolic activity even after 6 days of treatment. We assume that this enhanced metabolism is not compulsorily caused by an enhanced proliferation. More likely, the expressed cytokines might boost the cellular metabolism. ME-CFs on the other side demonstrated a much more enhanced metabolic activity already after 4 days of growth. We suspected that this increase in metabolism was not solely explainable by autocrine action of cytokines affecting cellular metabolism, but has its source in an increased autocrine stimulation by growth factors causing an enhanced proliferation of ME-CFs. In accordance to this, the proliferation assay showed a significantly increased proliferation of ME-CFs challenged with LPS, while ME-CSCs as well as ACFs did not display enhanced mitotic activity upon LPS stimulation (Fig. 4b and Additional file 2: Fig. S2 respectively). This might be explained by the fact, that IGF-2, TGF-β1 and bFGF, which were induced by LPS stimulation in ME-CFs (cf. Figures 2, 6 and Additional file 3: Fig. S3), stimulate the proliferation of fibroblasts [47]. Interestingly, TGF-β1also can induce proliferation via induction of bFGF expression in an autocrine fashion which further accelerates the proliferation of ME-CFs [48]. Furthermore, the proliferation of ME-CFs can also be induced by a secretion of EGF [49, 50] or EREG [51, 52] in LPS-stimulated ME-CFs (cf. Figures 2 and 6). Besides the self evident induction of proliferation by growth factors, the expression of cytokines might also play a role. Different studies demonstrated the increased proliferation in fibroblast by cytokines like IL-1α [53], IL-6 [54, 55], GM-CSF [56], IL-1β and TNF-α [57] (Fig. 6). Importantly, we were able to reduce the enhanced proliferation significantly by antagonistic blockage of TLR4 by LPS-RS. This effect is in accordance to the observed reduced expression of cytokines and growth factors upon LPS-RS treatment (Additional file 3: Fig. S3). Furthermore, we suggest that in cholesteatoma tissue the secreted growth factors and inflammatory mediators will additionally induce the hyperproliferation of keratinocytes (Fig. 6), the major symptom off cholesteatoma disease. Various studies demonstrated that the growth factors KGF [58], HGF [59], IGF-2, EGF [60], upregulated in LPS stimulated ME-CFs (Fig. 2), are known promoters of epidermal proliferation. We conclude that ME-CFs not only promote their own but rather the hyperproliferative character of the whole cholesteatoma tissue by secreting various growth factors and inflammatory mediators in vivo and that this route of pathogenesis is amplified by the high concentrations of LPS and DAMPs found in cholesteatoma tissue.

The major clinical picture of cholesteatoma disease is the relentless formation of keratinizing squamous epithelium. It is Important to mention that HGF [59] and KGF [58] and GM-CSF [61] are known promoters of epidermal differentiation. Previous studies demonstrated that the ME-CSCs are able to differentiate into the epidermal linage by KGF, EGF, IGF-2 and HGF [14]. Exactly these growth factors and GM-CSF where highly expressed and further upregulated upon LPS stimulation in ME-CFs. To examine if ME-CSCs can be considered as source of the self-renewal capacity of cholesteatoma tissue, we established an indirect co-culture of ME-CFs with ME-CSCs and stimulated this culture with LPS. Indeed, we could observe a strong upregulation of the cytokeratins 14, cytokeratins 16, cytokeratins 18 and cytokeratins 19 on mRNA level and cytokeratins 16 and cytokeratins 19 on protein level in ME-CSCs co-cultured with stimulated ME-CFs, while no such expression could be detected in co-culture of unstimulated ME-CFs and controls. Previous studies have shown that ME-CFs are able to increase epidermal differentiation in human keratinocyte cell lines [62] and that this effect is caused by KGF [38]. Intriguingly, KGF expression enables the development of cholesteatoma in an in vivo model [63]. We suggest that the epidermal differentiation of ME-CSCs by paracrine signalling of LPS treated ME-CFs resembles parts of cholesteatoma pathogenesis and more importantly its recurrence after incomplete surgical eradication [64] of cholesteatoma tissue and ME-CSCs respectively. Beyond this, our data permits the assumption, that the incomplete prevention of post operative inflammation is the main source of this route to recurrence. Interestingly, also middle ear epithelium can differentiate into stratified squamous epithelium showing keratinization upon induction of chronic otitis media in a rat model [65]. In addition to their epidermal differentiation, ME-CSCs showed a significantly enhanced expression of Ki-67 when co-cultured with LPS-treated ME-CFs. We assume that the expression of different growth factors in ME-CFs also supports the mitotic activity in ME-CSCs.

## Conclusion

Taken our experimental results together, the high recurrence upon infection of cholesteatoma [34] might be supported by an enhanced proliferation of ME-CFs and the increased epidermal differentiation of ME-CSCs upon paracrine stimulation of ME-CFs both caused upon TLR4 stimulation. Importantly, we found the TLR4 signalling reacts much more sensitive upon LPS stimulation in ME-CSCs and ME-CFs compared to ACSCs and ACFs resulting in the pathological inflammatory state in cholesteatoma tissue. Interestingly, LPS is by far not the only way to activate TLR4 signalling in cholesteatoma tissue. TLR4 signalling can also be induced by the DAMPs abundant in cholesteatoma tissue e.g. high-mobility group box 1 proteins (HMGB1) [66], Tenascin [67], fibronectin [5], S100A8, S100A9 [68] and also HSP60 and HSP70 [69]. Interestingly, the DAMPs HMGB1 and Tenascin are also suspected to contribute to cholesteatoma pathogenesis [66, 70]. We assume that pathogenesis as well as recurrence of cholesteatoma tissue upon TLR4 signalling can also be initiated by a non-infectious inflammatory response following tissue injury abundant in cholesteatoma.

Up to now there are several in vitro approaches to investigate possible ways to reduce the chance of cholesteatoma recurrence. Unfortunately, all of them focused solely on reducing the already triggered hyperproliferative behaviour of cholesteatoma epithelial cells. Arriaga et al*.* reduced the proliferation of keratinocytes by applying antibodies against the cholesteatoma-associated marker cytokeratin 10 [71]. Gluth and colleagues induced apoptosis in cholesteatoma-derived keratinocytes utilizing immunotargeted photodynamic therapy against the EGF receptor [72]. A study of Kara et al*.* demonstrated the induction of apoptosis in a cell culture model involving keratinocytes and fibroblasts by diclofenacsodium [73] and Jun et al. demonstrated that taraxerol induce apoptosis by inhibition of NF-κB signalling in epithelial cholesteatoma cells. An in vivo study on a chinchilla model showed a reduction of cholesteatoma development upon topical treatment with the cytostatic 5-fluorouracil [74]. This led to clinical application of 5-fluorouracil resulting in a reduction of recurrence upon topical application subsequent to surgery [75, 76] caused by the reduction of the proliferative activity of epithelial cells [77].

According to the data derived in this study, an inhibition of TLR4 signalling might bea promising treatment option. But in contrast to the approaches mentioned above, this will prevent the inflammation that causes cholesteatoma recurrence in the first place instead of dealing with its ensuing symptoms. In vivo experiments where already undertaken showing reduced recurrence in a gerbil model by application of a combination of antibiotics and hydrocortisone [78]. Indeed, it would make sense to interfere directly with TLR4 signalling most upstream with an antagonistic approach. In this study we applied LPS-RS to demonstrate this curative approach. However, other systemically applied antagonists were already investigated in clinical studies, e.g. Eritoran [79] or TAK-242 [80]. We propose that the topical application of these non-cytotoxic substances subsequent to surgery, maybe even as a bioabsorbable gel, might significantly reduce the recurrence of cholesteatoma. After a validation of these antagonists in terms of their ototoxicity in animal models, this should be further investigated on patients in clinical trials.

## Supplementary Information

**Additional file 1: Fig. S1.** Stimulation of ME-CFs with decreasing concentrations of LPS. A strong decrease in expression levels takes place between 1 µg/ml and 10 ng/ml. Between 1ng/ml and 0 ng/ml only insignificant changes can be observed (depicted: mean and standard deviation; unpaired two tailed t-test with Welch correction, 95% confidence interval, upon passed Shapiro-Wilk normality test, ns≥0.05).

**Additional file 2: Fig. S2.** Proliferation assay of ACFs with the addition of LPS. In contrast to ME-CFs no increase in proliferation can be detected in ACFs upon stimulation with LPS.

**Additional file 3: Fig. S3.** RT-qPCR data of transcripts responsible for fibroblast proliferation in ME-CFs stimulated with LPS and LPS-RS. All transcripts exhibit a certain upregulation upon stimulation with LPS. This upregulation can be reduced to at least the initial transcription level of ME-CFs under standard culture conditions.

## Data Availability

The datasets used and/or analysed during the current study are available from the corresponding author on reasonable request.

## Abbreviations

TLR4: Toll like receptor 4
LPS: Lipopolysaccharide
PAMP: Pathogen-associated molecular pattern
DAMP: Damage associated molecular Pattern
DMEM: Dulbecco’s Modified Eagle’s Medium
H&E: Haematoxylin and eosin
PFA: Paraformaldehyde
ME-CSC: Stem cell isolated from cholesteatoma tissue
ACSC: Stem cell isolated from auditory canal skin
SC-medium: Stem cell medium
DMEM/F12: Dulbeccos’s Modified Eagle Medium Nutrient Mixture F12
bFGF: Basic fibroblast growth factor
EGF: Human epidermal growth factor
ME-CF: Fibroblast from cholesteatoma tissue
ACF: Fibroblast from auditory canal skin
FB-medium: Fibroblast medium
LPS-RS: LPS from Rhodobacter sphaeroides
GAPDH: Glyceraldehyde 3-phosphate dehydrogenase
OM: Orders of magnitude
HMGB1: High-mobility group box 1 proteins
