# Supplementary figures and images for: Chronic inflammation of middle ear cholesteatoma promotes its recurrence via a paracrine mechanism
Source: Cell Commun Signal. 2021 Feb 24;19:25. doi: 10.1186/s12964-020-00690-y (PMC7903614; doi:10.1186/s12964-020-00690-y)

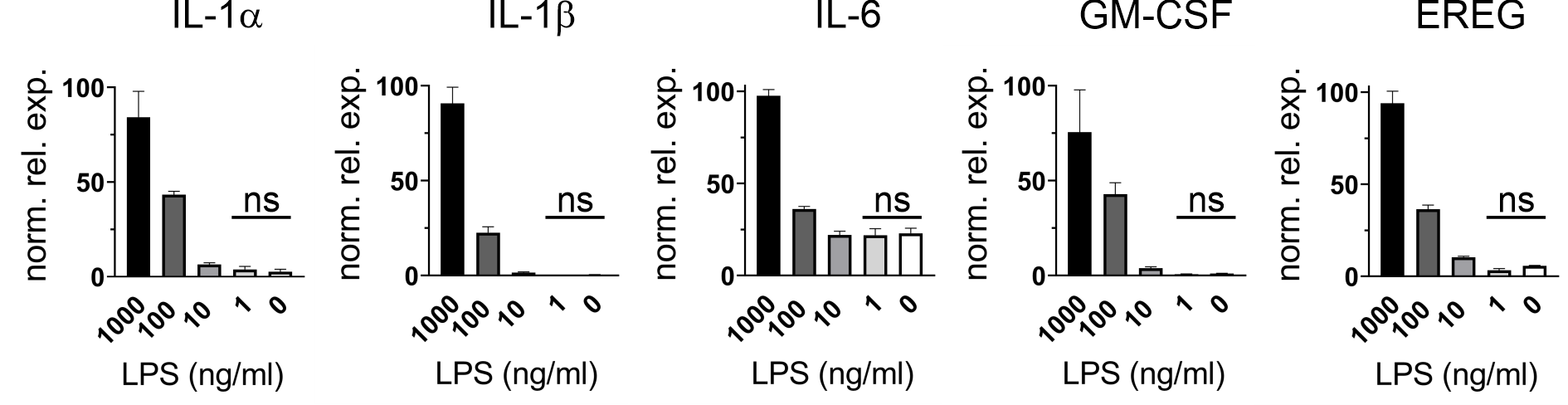

Supplement: Supplementary file 2 — Additional file 1: Fig. S1. Stimulation of ME-CFs with decreasing concentrations of LPS. A strong decrease in expression levels takes place between 1 µg/ml and 10 ng/ml. Between 1ng/ml and 0 ng/ml only insignificant changes can be observed (depicted: mean and standard deviation; unpaired two tailed t-test with Welch correction, 95% confidence interval, upon passed Shapiro-Wilk normality test, ns≥0.05). [file 12964_2020_690_MOESM2_ESM.tif]

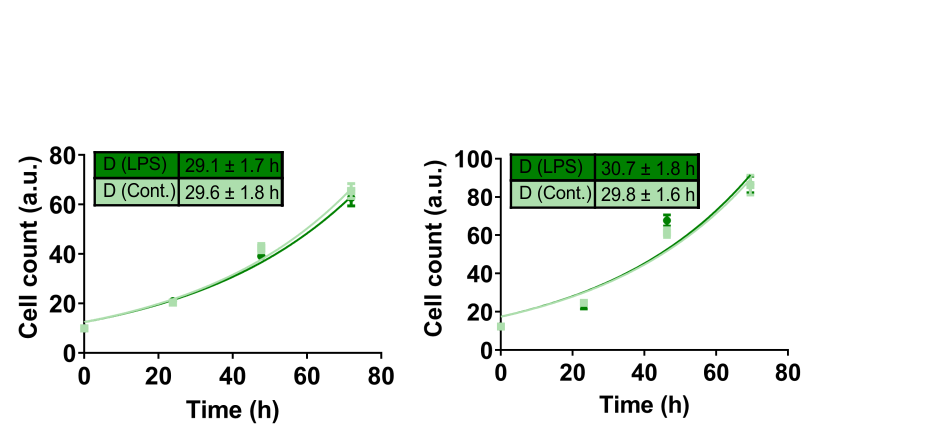

Supplement: Supplementary file 3 — Additional file 2: Fig. S2. Proliferation assay of ACFs with the addition of LPS. In contrast to ME-CFs no increase in proliferation can be detected in ACFs upon stimulation with LPS. [file 12964_2020_690_MOESM3_ESM.tif]

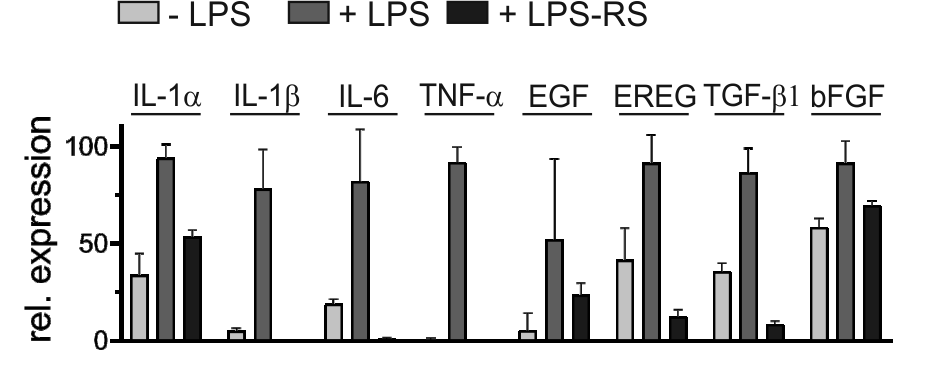

Supplement: Supplementary file 4 — Additional file 3: Fig. S3. RT-qPCR data of transcripts responsible for fibroblast proliferation in ME-CFs stimulated with LPS and LPS-RS. All transcripts exhibit a certain upregulation upon stimulation with LPS. This upregulation can be reduced to at least the initial transcription level of ME-CFs under standard culture conditions. [file 12964_2020_690_MOESM4_ESM.tif]
